# *catena*-Poly[[[diaqua­(di-2-pyridylamine-κ^2^ *N*,*N*′)nickel(II)]-μ-fumarato-κ^2^ *O* ^1^:*O* ^4^] tetra­hydrate]

**DOI:** 10.1107/S1600536810012225

**Published:** 2010-04-10

**Authors:** Anna Pavlová, Juraj Černák, Klaus Harms

**Affiliations:** a Department of Inorganic Chemistry, Institute of Chemistry, P. J. Šafárik University, Moyzesova 11, 041 54 Košice, Slovakia; b Fachbereich Chemie der Philipps Universität, Hans-Meerwein Strasse, D-35032 Marburg, Germany

## Abstract

In the crystal structure of the title compound, {[Ni(C_4_H_2_O_4_)(C_10_H_9_N_3_)(H_2_O)_2_]·4H_2_O}_*n*_, zigzag chains are built up from *cis*-[Ni(dpya)(H_2_O)_2_]^2+^ cations (dpya is di-2-pyridylamine) linked by bis-monodentate coordinated bridging fumarate ligands. The Ni^II^ atom is coordinated by one chelating dpya ligand, two aqua ligands in *trans* positions and two monodentate fumarate ligands in *cis* positions in the form of a deformed octa­hedron. The water mol­ecules, O atoms of the fumarate carboxyl­ate groups and the amine group of the dpya ligand are involved in an extended network of intra- and inter­molecular O—H⋯O hydrogen bonds. Moreover, π–π inter­actions between the pyridine rings of the dpya ligand contribute to the stability of the structure. Two of the five uncoordinated water molecules are half-occupied.

## Related literature

Several crystal structures of Ni^II^ fumarato (fum) complexes with bridging fumarato ligands have been reported in the literature, e.g. [Ni_2_(*phen*)_4_(fumarate)(H_2_O)_2_]fumarate·16H_2_O (*phen* = 1,10-phenantroline) (Ma *et al.*, 2003 ▶) with a dinuclear structure, [Ni(*py*)_3_(fumarate)_2_]·*py* (*py*= pyridine (Mori *et al.*, 2004 ▶) and [Ni(fumarate)(H_2_O)_4_] (Xie *et al.*, 2003 ▶), both forming chain-like structures, or [Ni(phen)fum)] exhibiting a two-dimensional structure (Černák *et al.*, 2009 ▶). For structurally characterized complexes of Ni^II^ containing the dpya ligand (dpya = 2,2′-dipyridylamine), see, for example: [Ni(dpya)(*ox*)]_*n *_(ox = oxalato) (Lu *et al.*, 2001 ▶) or [Ni(dpya)_2_(*dca*)_2_] (*dca* = dicyanamidato) complexes (Huang *et al.*, 2006 ▶).
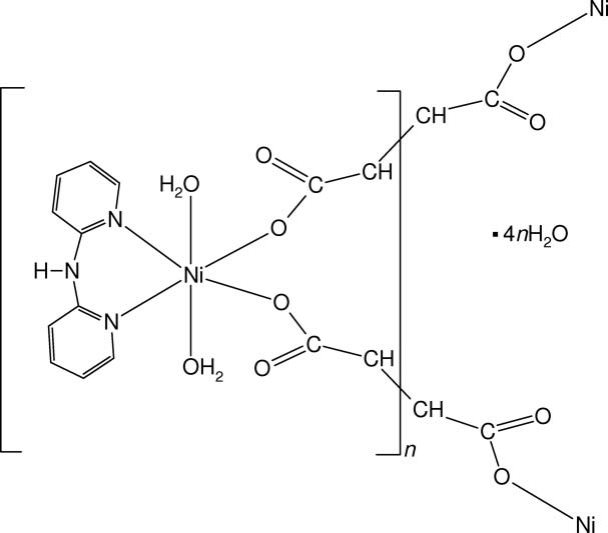


## Experimental

### 

#### Crystal data

[Ni(C_4_H_2_O_4_)(C_10_H_9_N_3_)(H_2_O)_2_]·4H_2_O
*M* *_r_* = 450.03
Monoclinic, 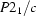

*a* = 12.1421 (12) Å
*b* = 12.4034 (8) Å
*c* = 12.8701 (13) Å
β = 96.138 (12)°
*V* = 1927.2 (3) Å^3^
*Z* = 4
Mo *K*α radiation
μ = 1.06 mm^−1^
*T* = 193 K
0.42 × 0.36 × 0.16 mm

#### Data collection

Stoe IPDS diffractometer
Absorption correction: gaussian (*WinGX*; Farrugia, 1999 ▶) *T*_min_ = 0.750, *T*_max_ = 0.836
13672 measured reflections
3397 independent reflections
2538 reflections with *I* > 2σ(*I*)
*R*_int_ = 0.048

#### Refinement

*R*[*F* ^2^ > 2σ(*F* ^2^)] = 0.029
*wR*(*F* ^2^) = 0.069
*S* = 0.90
3397 reflections
280 parameters
16 restraints
H atoms treated by a mixture of independent and constrained refinement
Δρ_max_ = 0.42 e Å^−3^
Δρ_min_ = −0.31 e Å^−3^

### 

Data collection: *IPDS* (Stoe & Cie, 1996 ▶); cell refinement: *IPDS*; data reduction: *IPDS*; program(s) used to solve structure: *SHELXS97* (Sheldrick, 2008 ▶); program(s) used to refine structure: *SHELXL97* (Sheldrick, 2008 ▶); molecular graphics: *DIAMOND* (Brandenburg, 2006 ▶); software used to prepare material for publication: *SHELXL97*.

## Supplementary Material

Crystal structure: contains datablocks I, global. DOI: 10.1107/S1600536810012225/hg2662sup1.cif

Structure factors: contains datablocks I. DOI: 10.1107/S1600536810012225/hg2662Isup2.hkl

Additional supplementary materials:  crystallographic information; 3D view; checkCIF report

## Figures and Tables

**Table 1:** Hydrogen-bond geometry (Å, °)

*D*—H⋯*A*	*D*—H	H⋯*A*	*D*⋯*A*	*D*—H⋯*A*
O5—H5*A*⋯O4^i^	0.85	1.87	2.713 (2)	175
O5—H5*B*⋯O11^ii^	0.85	2.01	2.859 (3)	177
O6—H6*B*⋯O2	0.85	1.88	2.693 (2)	161
O6—H6*A*⋯O4	0.85	1.88	2.706 (2)	165
O7—H7*B*⋯O11^iii^	0.85	2.32	2.988 (4)	136
O7—H7*A*⋯O9^ii^	0.85	1.94	2.700 (7)	148
O10—H10*B*⋯O2^iii^	0.85	1.98	2.777 (3)	155
O10—H10*A*⋯O8	0.85	2.36	2.987 (6)	131
O10—H10*A*⋯O9	0.85	1.83	2.588 (6)	147
O11—H11*A*⋯O5^iv^	0.85	2.43	3.116 (3)	138
O11—H11*B*⋯O10	0.85	2.14	2.870 (4)	144
N3—H3*N*⋯O7^v^	0.89 (1)	2.03 (1)	2.917 (3)	175 (3)

Symmetry codes: (i) 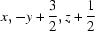; (ii) 
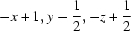
; (iii) 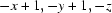; (iv) 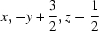; (v) 
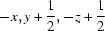
.

**Fig. 1.**
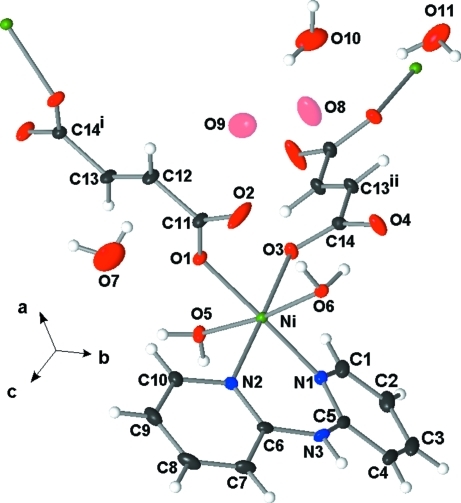
View of the crystal structure 1. The thermal ellipsoids are drawn at 30 % probability level. Symmetry codes: i: 1 - x, -0.5 + y, 0.5 - z, ii: 1 - x, 0.5 + y, 0.5 - z.

**Fig. 2.**
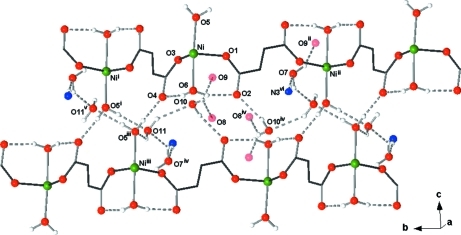
Chain-like structure of 1 along with intramolecular and intermolecular hydrogen bonds (dashed lines). The bonds within the chain propagation are dark grey. The oxygen atoms with half ocupancy are drawn as half-transparent balls. Symmetry codes: i: 1 - x, 0.5 + y, 0.5 - z; ii: 1 - x, -0.5 + y, 0.5 - z; iii: x, 1.5 - y, -0.5 - z; iv: 1 - x, 1 - y, - z; v: 1 - x, 2 - y, - z; vi: - x, -0.5 + y, 0.5 - z.

**Fig. 3.**
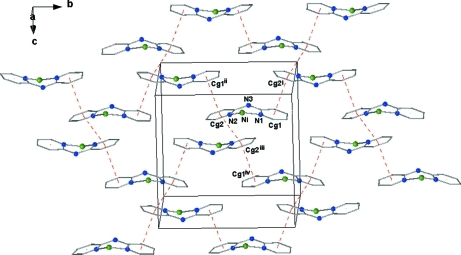
Scheme of π-π-interactions in 1. For the sake of clarity only the {Ni(dpya)} structural units are shown without hydrogen atoms. Symmetry codes: i: - x, 0.5 + y, 0.5 - z; ii: - x, -0.5 + y, 0.5 - z; iii: -x, 1 - y, 1 - z; iv: x, 1.5 - y, 0.5 + z.

## supplementary crystallographic information

### Comment

As a continuation of our studies on syntheses, crystal structures and relationship structure vs. magnetic properties of low-dimensional magnetic materials [Černák *et al.*, 2009] we have occasionally isolated a crystal of the title compound 1. Its crystal structure is polymeric and is composed of zig–zag chains and water molecules of crystallization (Fig. 1, 2, 3). The chains are formed by octahedrally coordinated Ni^II^ atoms linked by two bis(monodentate) fumarato ligands (Fig. 1, 2). Similar chain-like structure was observed in an another fumarato complex, [Ni(*py*)_3_(fumarate)_2_].*py* (*py*= pyridine) in which the Ni^II^ atoms are bridged alternatively by bis(monodentate) and bis(bidentate) bonded fumarato ligands [Mori *et al.*, 2004].

The heteroleptic coordination sphere of the Ni^II^ atom beside two fumarato ligands is completed by one bidentate chelate bonded dpya ligand and two aqua ligands placed in *trans* positions (Fig. 1). As can be seen from the values of the bond angles (Table 2), the octahedron around the Ni^II^ atom is somewhat deformed. The mean Ni–N bond lengths is 2.059 (3) Å, and the Ni–O bond lengths exhibit values from the range 2.048 (2) – 2.099 (2) Å. Similar values of Ni–N and Ni–O bond distances were observed in complexes [Ni(dpya)(*ox*)]*_n_* (Ni–N: 2.046 (2) Å) (Lu *et al.*, 2001) and [Ni(fumarate)(H_2_O)_4_] (Ni–O: 2.064 (2) Å) (Xie *et al.*, 2003).

The same type of bridging formed by bis(monodentato) fumarate ligand was already observed in dinuclear [Ni_2_(*phen*)_4_(fumarate)(H_2_O)_2_]fumarate.16H_2_O complex (*phen* = 1,10-phenantroline) (Ma *et al.*, 2003). The observed geometric parameters associated with the fumarate ligand in 1 are similar to those found in the previously mentioned dinuclear complex (Ma *et al.*, 2003) or in ionic.

The geometric parameters associated with dpya and aqua ligands are normal [Lu *et al.*, 2001; Huang *et al.*, 2006]. There are five general crystallographically distinct positions in the unit cell occupied by not coordinated water molecules. Among these two positions (O8 and O9) are partially occupied with s.o.f. put to half as required by proximity of symmetry (-1) related positions of O8 and proximity of the O9 water oxygen to O8, respectively. The water molecules along with the not coordinated oxygen atoms from carboxylate groups are involved in hydrogen bonds of the O—H···O type; some of these HBs are intramolecular (Fig. 2, Table 3). In the hydrogen bonding system is involved also the dpyaligand through N—H···O type hydrogen bond (Fig. 2, Table 3). Between pairs of dpya ligands π–π interactions operate which further stabilize the structure (Fig. 3). The *C*g1···*C*g2^i^ distance (symmetry code (i) -*x*, 0.5+y, 0.5-*z*, where *Cg*1 and *Cg*2 are centroids of the rings (N1/C1—C5) and (N2/C6—C10), respectively) between the aromatic rings is 3.723 (1) Å; these interactions links the {Ni(dpya)} units into layers lying in the *bc* plane.

### Experimental

With the exception of dpya, which was of *purum* quality, the other reagents were of analytical grade and all were used without further purification. The title complex was prepared using the following procedure. An aqueous solution of Ni(CH_3_COO)_2_.4H_2_O(248 mg, 1 mmol in 30 cm^3 ^H_2_O) and a solution of 171 mg (1 mmol) dpya ligand in 40 cm^3^of EtOH (96 %vv) were mixed firstly. To the formed azure hot (90 °C) solution solid fumaric acid (116 mg, 1 mmol) and aqueous solution of NaOH (2 cm^3^, 1 M) were added successively and the reaction mixture was stirred 60 minutes at 90 °C. The formed blue solution was left to evaporate slowly at room temperature. Within a week, in one of several reaction attempts, few blue plates of the title compound appeared. One crystal was picked off for X-ray structure analysis. After disturbing the mother liquor immediate jellification started which prevented isolation of further crystals.

### Refinement

All H atoms linked to aromatic carbon atoms were positioned geometrically, with C–H = 0.96Å and refined as riding with *U*iso(H) = 1.2*U*eq(C). The hydrogen atom H3N bonded to amine nitrogen atom N3 was refined with restrained distance N—H 0.89 Å and with *U*_iso_(H) = 1.2*U*_eq_(N). The hydrogen atoms from water molecules with full oxygen atom occupancies were located using the CALC-OH program within WinGX package (Farrugia, 1999) and refined with constrained geometric parameters (O—H, 0.85 Å and H···H, 1.334 Å); their thermal parameters were tied with parent oxygen atom(*U*_iso_(H) = 1.5*U*_eq_(O)).

### Crystal data

**Table:**

[Ni(C_4_H_2_O_4_)(C_10_H_9_N_3_)(H_2_O)_2_]·4H_2_O	*F*(000) = 936
*M**_r_* = 450.03	*D*_x_ = 1.551 Mg m^−^^3^
Monoclinic, *P*2_1_/*c*	Mo *K*α radiation, λ = 0.71073 Å
Hall symbol: -P 2ybc	Cell parameters from 8000 reflections
*a* = 12.1421 (12) Å	θ = 2.3–25.0°
*b* = 12.4034 (8) Å	µ = 1.06 mm^−^^1^
*c* = 12.8701 (13) Å	*T* = 193 K
β = 96.138 (12)°	Plates, blue
*V* = 1927.2 (3) Å^3^	0.42 × 0.36 × 0.16 mm
*Z* = 4	

### Data collection

**Table:**

Stoe IPDS diffractometer	3397 independent reflections
Radiation source: fine-focus sealed tube	2538 reflections with *I* > 2σ(*I*)
graphite	*R*_int_ = 0.048
φ scans	θ_max_ = 25.0°, θ_min_ = 2.3°
Absorption correction: gaussian (*WinGX*; Farrugia, 1999)	*h* = −14→14
*T*_min_ = 0.750, *T*_max_ = 0.836	*k* = −14→14
13672 measured reflections	*l* = −15→15

### Refinement

**Table:**

Refinement on *F*^2^	Primary atom site location: structure-invariant direct methods
Least-squares matrix: full	Secondary atom site location: difference Fourier map
*R*[*F*^2^ > 2σ(*F*^2^)] = 0.029	Hydrogen site location: inferred from neighbouring sites
*wR*(*F*^2^) = 0.069	H atoms treated by a mixture of independent and constrained refinement
*S* = 0.90	*w* = 1/[σ^2^(*F*_o_^2^) + (0.0432*P*)^2^] where *P* = (*F*_o_^2^ + 2*F*_c_^2^)/3
3397 reflections	(Δ/σ)_max_ = 0.001
280 parameters	Δρ_max_ = 0.42 e Å^−^^3^
16 restraints	Δρ_min_ = −0.31 e Å^−^^3^

### Special details

**Table:**

Experimental. Absorption correction: a grid of 8 x 8 x 8 = 512 sampling points was used
Geometry. All esds (except the esd in the dihedral angle between two l.s. planes) are estimated using the full covariance matrix. The cell esds are taken into account individually in the estimation of esds in distances, angles and torsion angles; correlations between esds in cell parameters are only used when they are defined by crystal symmetry. An approximate (isotropic) treatment of cell esds is used for estimating esds involving l.s. planes.
Refinement. Refinement of *F*^2^ against ALL reflections. The weighted *R*-factor wR and goodness of fit *S* are based on *F*^2^, conventional *R*-factors *R* are based on *F*, with *F* set to zero for negative *F*^2^. The threshold expression of *F*^2^ > σ(*F*^2^) is used only for calculating *R*-factors(gt) etc. and is not relevant to the choice of reflections for refinement. *R*-factors based on *F*^2^ are statistically about twice as large as those based on *F*, and *R*- factors based on ALL data will be even larger.

### Fractional atomic coordinates and isotropic or equivalent isotropic displacement parameters (Å^2^)

**Table:**

	*x*	*y*	*z*	*U*_iso_*/*U*_eq_	Occ. (<1)
Ni	0.24621 (2)	0.59939 (2)	0.31901 (2)	0.01368 (9)	
N1	0.15403 (15)	0.73037 (15)	0.35699 (14)	0.0191 (4)	
N2	0.12272 (15)	0.50121 (15)	0.36286 (14)	0.0179 (4)	
N3	−0.01512 (15)	0.63520 (17)	0.32761 (16)	0.0237 (5)	
H3N	−0.0882 (3)	0.644 (2)	0.317 (2)	0.028*	
C1	0.2063 (2)	0.82458 (19)	0.38251 (18)	0.0237 (5)	
H1	0.2833	0.8248	0.3912	0.028*	
C2	0.1523 (2)	0.9199 (2)	0.3963 (2)	0.0310 (6)	
H2	0.1917	0.9827	0.4141	0.037*	
C3	0.0372 (2)	0.9198 (2)	0.3831 (2)	0.0336 (7)	
H3	−0.0020	0.9835	0.3898	0.040*	
C4	−0.0179 (2)	0.8252 (2)	0.35992 (19)	0.0286 (6)	
H4	−0.0949	0.8236	0.3519	0.034*	
C5	0.04309 (19)	0.73020 (19)	0.34832 (18)	0.0204 (5)	
C6	0.01564 (18)	0.53059 (19)	0.35449 (18)	0.0197 (5)	
C7	−0.0681 (2)	0.4572 (2)	0.37210 (19)	0.0278 (6)	
H7	−0.1419	0.4787	0.3649	0.033*	
C8	−0.0401 (2)	0.3536 (2)	0.4000 (2)	0.0321 (6)	
H8	−0.0949	0.3038	0.4111	0.039*	
C9	0.0702 (2)	0.3232 (2)	0.4115 (2)	0.0298 (6)	
H9	0.0909	0.2535	0.4318	0.036*	
C10	0.14803 (19)	0.3986 (2)	0.39222 (18)	0.0232 (5)	
H10	0.2221	0.3782	0.3997	0.028*	
C11	0.35852 (19)	0.41929 (18)	0.21127 (18)	0.0211 (5)	
C12	0.4552 (2)	0.34435 (19)	0.2153 (2)	0.0237 (5)	
H12	0.4647	0.3038	0.1561	0.028*	
C13	0.52676 (19)	0.33281 (19)	0.29733 (19)	0.0219 (5)	
H13	0.5169	0.3725	0.3569	0.026*	
C14	0.37611 (18)	0.75874 (18)	0.20000 (18)	0.0194 (5)	
O1	0.34712 (13)	0.47257 (12)	0.29159 (12)	0.0215 (4)	
O2	0.29572 (18)	0.42549 (19)	0.12676 (16)	0.0551 (7)	
O3	0.37265 (13)	0.69567 (13)	0.27580 (13)	0.0232 (4)	
O4	0.30434 (14)	0.76425 (15)	0.12189 (13)	0.0321 (4)	
O5	0.33762 (13)	0.59538 (13)	0.46659 (12)	0.0219 (3)	
H5A	0.3240	0.6366	0.5164	0.033*	
H5B	0.3568	0.5360	0.4962	0.033*	
O6	0.17160 (12)	0.59825 (13)	0.16421 (12)	0.0175 (3)	
H6A	0.2057	0.6510	0.1403	0.026*	
H6B	0.2005	0.5428	0.1393	0.026*	
O7	0.25446 (19)	0.1566 (3)	0.2196 (3)	0.0818 (9)	
H7A	0.3002	0.1105	0.2475	0.123*	
H7B	0.2680	0.1597	0.1562	0.123*	
O8	0.4461 (4)	0.5742 (5)	0.0058 (5)	0.0810 (18)	0.50
O9	0.5592 (5)	0.5821 (4)	0.1705 (5)	0.0784 (17)	0.50
O10	0.6567 (2)	0.6997 (2)	0.04168 (19)	0.0643 (7)	
H10B	0.6851	0.6769	−0.0117	0.096*	
H10A	0.6224	0.6455	0.0627	0.096*	
O11	0.58881 (17)	0.89674 (18)	−0.0632 (2)	0.0576 (6)	
H11A	0.5219	0.8939	−0.0903	0.086*	
H11B	0.5919	0.8526	−0.0124	0.086*	

### Atomic displacement parameters (Å^2^)

**Table:**

	*U*^11^	*U*^22^	*U*^33^	*U*^12^	*U*^13^	*U*^23^
Ni	0.01153 (14)	0.01349 (14)	0.01623 (15)	0.00042 (12)	0.00244 (10)	0.00048 (13)
N1	0.0205 (10)	0.0199 (10)	0.0169 (10)	0.0030 (8)	0.0016 (8)	0.0003 (8)
N2	0.0179 (9)	0.0190 (10)	0.0168 (10)	−0.0008 (8)	0.0028 (8)	0.0003 (8)
N3	0.0118 (9)	0.0314 (11)	0.0281 (11)	0.0050 (8)	0.0038 (8)	0.0010 (9)
C1	0.0292 (13)	0.0187 (12)	0.0225 (13)	0.0014 (10)	−0.0008 (10)	−0.0014 (10)
C2	0.0468 (16)	0.0206 (13)	0.0245 (13)	0.0045 (11)	−0.0013 (12)	−0.0031 (11)
C3	0.0504 (17)	0.0292 (15)	0.0216 (13)	0.0210 (12)	0.0059 (12)	0.0001 (11)
C4	0.0291 (13)	0.0361 (15)	0.0209 (13)	0.0158 (12)	0.0033 (11)	0.0010 (12)
C5	0.0200 (12)	0.0263 (13)	0.0152 (11)	0.0073 (10)	0.0034 (9)	0.0029 (10)
C6	0.0165 (11)	0.0283 (13)	0.0151 (11)	−0.0024 (10)	0.0046 (9)	−0.0017 (10)
C7	0.0182 (12)	0.0417 (16)	0.0241 (13)	−0.0099 (11)	0.0052 (10)	−0.0056 (12)
C8	0.0348 (15)	0.0390 (15)	0.0239 (14)	−0.0195 (13)	0.0088 (11)	−0.0038 (12)
C9	0.0435 (16)	0.0223 (13)	0.0244 (14)	−0.0089 (11)	0.0079 (12)	0.0034 (11)
C10	0.0255 (12)	0.0232 (12)	0.0211 (12)	−0.0002 (11)	0.0032 (10)	0.0036 (11)
C11	0.0189 (11)	0.0212 (13)	0.0230 (12)	0.0044 (9)	0.0004 (10)	−0.0053 (10)
C12	0.0246 (13)	0.0235 (13)	0.0231 (13)	0.0075 (10)	0.0032 (11)	−0.0082 (10)
C13	0.0211 (12)	0.0238 (12)	0.0212 (13)	0.0062 (10)	0.0038 (10)	−0.0070 (10)
C14	0.0178 (11)	0.0204 (12)	0.0205 (13)	−0.0017 (9)	0.0046 (10)	0.0007 (10)
O1	0.0214 (8)	0.0229 (9)	0.0204 (9)	0.0079 (7)	0.0028 (7)	−0.0021 (7)
O2	0.0490 (12)	0.0711 (16)	0.0394 (12)	0.0426 (11)	−0.0229 (10)	−0.0330 (11)
O3	0.0168 (8)	0.0257 (9)	0.0263 (9)	−0.0048 (7)	−0.0010 (7)	0.0096 (8)
O4	0.0302 (10)	0.0396 (11)	0.0245 (10)	−0.0173 (8)	−0.0065 (8)	0.0124 (8)
O5	0.0287 (9)	0.0184 (8)	0.0177 (8)	0.0061 (8)	−0.0018 (7)	−0.0021 (7)
O6	0.0152 (7)	0.0167 (7)	0.0208 (8)	−0.0001 (7)	0.0022 (6)	−0.0009 (7)
O7	0.0263 (12)	0.090 (2)	0.127 (3)	−0.0084 (13)	−0.0006 (15)	0.001 (2)
O8	0.050 (3)	0.098 (4)	0.093 (4)	−0.020 (3)	0.002 (3)	0.052 (3)
O9	0.079 (4)	0.048 (3)	0.115 (5)	0.000 (3)	0.043 (3)	0.012 (3)
O10	0.0457 (14)	0.100 (2)	0.0457 (14)	0.0087 (14)	−0.0023 (10)	−0.0358 (15)
O11	0.0310 (11)	0.0421 (13)	0.096 (2)	−0.0019 (10)	−0.0092 (11)	−0.0267 (13)

### Geometric parameters (Å, °)

**Table:**

Ni—O1	2.0475 (15)	C8—H8	0.9300
Ni—N2	2.0569 (18)	C9—C10	1.371 (3)
Ni—N1	2.0611 (19)	C9—H9	0.9300
Ni—O3	2.0676 (15)	C10—H10	0.9300
Ni—O5	2.0955 (16)	C11—O1	1.247 (3)
Ni—O6	2.0986 (16)	C11—O2	1.262 (3)
N1—C5	1.340 (3)	C11—C12	1.493 (3)
N1—C1	1.353 (3)	C12—C13	1.302 (3)
N2—C6	1.343 (3)	C12—H12	0.9300
N2—C10	1.353 (3)	C13—C14^i^	1.492 (3)
N3—C6	1.384 (3)	C13—H13	0.9300
N3—C5	1.385 (3)	C14—O3	1.255 (3)
N3—H3N	0.8899 (10)	C14—O4	1.260 (3)
C1—C2	1.372 (3)	C14—C13^ii^	1.492 (3)
C1—H1	0.9300	O5—H5A	0.8499
C2—C3	1.390 (4)	O5—H5B	0.8499
C2—H2	0.9300	O6—H6A	0.8499
C3—C4	1.368 (4)	O6—H6B	0.8499
C3—H3	0.9300	O7—H7A	0.8499
C4—C5	1.408 (3)	O7—H7B	0.8500
C4—H4	0.9300	O10—H10B	0.8500
C6—C7	1.401 (3)	O10—H10A	0.8499
C7—C8	1.367 (4)	O11—H11A	0.8499
C7—H7	0.9300	O11—H11B	0.8500
C8—C9	1.384 (4)		
			
O1—Ni—N2	93.42 (7)	N2—C6—N3	120.5 (2)
O1—Ni—N1	175.24 (7)	N2—C6—C7	121.6 (2)
N2—Ni—N1	88.36 (8)	N3—C6—C7	117.9 (2)
O1—Ni—O3	85.53 (7)	C8—C7—C6	119.2 (2)
N2—Ni—O3	178.84 (7)	C8—C7—H7	120.4
N1—Ni—O3	92.65 (7)	C6—C7—H7	120.4
O1—Ni—O5	82.49 (6)	C7—C8—C9	119.7 (2)
N2—Ni—O5	93.92 (7)	C7—C8—H8	120.2
N1—Ni—O5	92.99 (7)	C9—C8—H8	120.2
O3—Ni—O5	85.46 (6)	C10—C9—C8	118.2 (2)
O1—Ni—O6	92.10 (6)	C10—C9—H9	120.9
N2—Ni—O6	90.18 (7)	C8—C9—H9	120.9
N1—Ni—O6	92.30 (7)	N2—C10—C9	123.5 (2)
O3—Ni—O6	90.35 (6)	N2—C10—H10	118.3
O5—Ni—O6	173.40 (6)	C9—C10—H10	118.3
C5—N1—C1	117.6 (2)	O1—C11—O2	124.9 (2)
C5—N1—Ni	123.07 (16)	O1—C11—C12	117.2 (2)
C1—N1—Ni	119.05 (15)	O2—C11—C12	117.9 (2)
C6—N2—C10	117.80 (19)	C13—C12—C11	123.4 (2)
C6—N2—Ni	123.03 (15)	C13—C12—H12	118.3
C10—N2—Ni	118.87 (15)	C11—C12—H12	118.3
C6—N3—C5	129.1 (2)	C12—C13—C14^i^	123.0 (2)
C6—N3—H3N	113.0 (18)	C12—C13—H13	118.5
C5—N3—H3N	114.0 (18)	C14^i^—C13—H13	118.5
N1—C1—C2	123.8 (2)	O3—C14—O4	125.2 (2)
N1—C1—H1	118.1	O3—C14—C13^ii^	117.3 (2)
C2—C1—H1	118.1	O4—C14—C13^ii^	117.5 (2)
C1—C2—C3	118.2 (2)	C11—O1—Ni	132.19 (15)
C1—C2—H2	120.9	C14—O3—Ni	131.06 (14)
C3—C2—H2	120.9	Ni—O5—H5A	122.8
C4—C3—C2	119.2 (2)	Ni—O5—H5B	121.3
C4—C3—H3	120.4	H5A—O5—H5B	104.5
C2—C3—H3	120.4	Ni—O6—H6A	99.3
C3—C4—C5	119.4 (2)	Ni—O6—H6B	102.1
C3—C4—H4	120.3	H6A—O6—H6B	104.5
C5—C4—H4	120.3	H7A—O7—H7B	104.5
N1—C5—N3	120.3 (2)	H10B—O10—H10A	104.5
N1—C5—C4	121.7 (2)	H11A—O11—H11B	104.5
N3—C5—C4	118.0 (2)		
			
N2—Ni—N1—C5	−30.95 (18)	C10—N2—C6—N3	178.2 (2)
O3—Ni—N1—C5	149.62 (18)	Ni—N2—C6—N3	−8.2 (3)
O5—Ni—N1—C5	−124.78 (18)	C10—N2—C6—C7	−2.0 (3)
O6—Ni—N1—C5	59.16 (18)	Ni—N2—C6—C7	171.57 (17)
N2—Ni—N1—C1	155.52 (18)	C5—N3—C6—N2	−30.8 (4)
O3—Ni—N1—C1	−23.91 (18)	C5—N3—C6—C7	149.4 (2)
O5—Ni—N1—C1	61.69 (18)	N2—C6—C7—C8	1.0 (4)
O6—Ni—N1—C1	−114.37 (17)	N3—C6—C7—C8	−179.2 (2)
O1—Ni—N2—C6	−154.26 (18)	C6—C7—C8—C9	0.7 (4)
N1—Ni—N2—C6	30.16 (18)	C7—C8—C9—C10	−1.3 (4)
O5—Ni—N2—C6	123.05 (18)	C6—N2—C10—C9	1.4 (3)
O6—Ni—N2—C6	−62.14 (18)	Ni—N2—C10—C9	−172.42 (19)
O1—Ni—N2—C10	19.27 (17)	C8—C9—C10—N2	0.2 (4)
N1—Ni—N2—C10	−156.31 (17)	O1—C11—C12—C13	−0.2 (4)
O5—Ni—N2—C10	−63.42 (17)	O2—C11—C12—C13	178.6 (3)
O6—Ni—N2—C10	111.39 (17)	C11—C12—C13—C14^i^	−179.2 (2)
C5—N1—C1—C2	−2.3 (3)	O2—C11—O1—Ni	−9.5 (4)
Ni—N1—C1—C2	171.57 (19)	C12—C11—O1—Ni	169.25 (15)
N1—C1—C2—C3	−0.2 (4)	N2—Ni—O1—C11	91.7 (2)
C1—C2—C3—C4	1.9 (4)	O3—Ni—O1—C11	−88.8 (2)
C2—C3—C4—C5	−1.1 (4)	O5—Ni—O1—C11	−174.8 (2)
C1—N1—C5—N3	−176.7 (2)	O6—Ni—O1—C11	1.4 (2)
Ni—N1—C5—N3	9.7 (3)	O4—C14—O3—Ni	−12.0 (4)
C1—N1—C5—C4	3.2 (3)	C13^ii^—C14—O3—Ni	168.21 (15)
Ni—N1—C5—C4	−170.46 (17)	O1—Ni—O3—C14	115.7 (2)
C6—N3—C5—N1	29.9 (4)	N1—Ni—O3—C14	−68.7 (2)
C6—N3—C5—C4	−150.0 (2)	O5—Ni—O3—C14	−161.5 (2)
C3—C4—C5—N1	−1.6 (4)	O6—Ni—O3—C14	23.6 (2)
C3—C4—C5—N3	178.3 (2)		

Symmetry codes: (i) −*x*+1, *y*−1/2, −*z*+1/2; (ii) −*x*+1, *y*+1/2, −*z*+1/2.

### Hydrogen-bond geometry (Å, °)

**Table:**

*D*—H···*A*	*D*—H	H···*A*	*D*···*A*	*D*—H···*A*
O5—H5A···O4^iii^	0.85	1.87	2.713 (2)	175
O5—H5B···O11^i^	0.85	2.01	2.859 (3)	177
O6—H6B···O2	0.85	1.88	2.693 (2)	161
O6—H6A···O4	0.85	1.88	2.706 (2)	165
O7—H7B···O11^iv^	0.85	2.32	2.988 (4)	136
O7—H7A···O9^i^	0.85	1.94	2.700 (7)	148
O10—H10B···O2^iv^	0.85	1.98	2.777 (3)	155
O10—H10A···O8	0.85	2.36	2.987 (6)	131
O10—H10A···O9	0.85	1.83	2.588 (6)	147
O11—H11A···O5^v^	0.85	2.43	3.116 (3)	138
O11—H11B···O10	0.85	2.14	2.870 (4)	144
N3—H3N···O7^vi^	0.89 (1)	2.03 (1)	2.917 (3)	175 (3)

Symmetry codes: (iii) *x*, −*y*+3/2, *z*+1/2; (i) −*x*+1, *y*−1/2, −*z*+1/2; (iv) −*x*+1, −*y*+1, −*z*; (v) *x*, −*y*+3/2, *z*−1/2; (vi) −*x*, *y*+1/2, −*z*+1/2.
